# In Situ Microstructure Modification Using a Layerwise Surface-Preheating Laser Scan of Ti-6Al-4V during Laser Powder Bed Fusion

**DOI:** 10.3390/ma17081929

**Published:** 2024-04-22

**Authors:** Ahmet Alptug Tanrikulu, Behzad Farhang, Aditya Ganesh-Ram, Hamidreza Hekmatjou, Sadman Hafiz Durlov, Amirhesam Amerinatanzi

**Affiliations:** 1 Materials Science and Engineering, The University of Texas at Arlington, Arlington, TX 76019, USA; axt5488@mavs.uta.edu; 2 Turkish Aerospace Industries, Ankara 06980, Türkiye; 3 Mechanical Engineering, The University of Texas at Arlington, Arlington, TX 76019, USA; behzad.farhang@mavs.uta.edu (B.F.); adityakrishna.ganeshram@mavs.uta.edu (A.G.-R.); hxh2477@mavs.uta.edu (H.H.); sxd5337@mavs.uta.edu (S.H.D.); 4 ArcelorMittal North America, East Chicago, IN 46312, USA

**Keywords:** in situ thermal processing, LPBF, Ti-6Al-4V, microstructure tailoring, layerwise preheating

## Abstract

An innovative in situ thermal approach in the domain of LPBF for Ti-6Al-4V fabrication has been carried out with results directing towards an improved fatigue life without the need for post-processing. The thermal process involves an additional laser scan with different process parameters to preheat the selected regions of each layer of the powder bed prior to their full melting. This preheating step influences the cooling rate, which in turn affects surface characteristics and subsurface microstructure, both of which are directly correlated with fatigue properties. A thorough analysis has been conducted by comparing the preheated samples with reference samples with no preheating. Without any additional thermal processing, the preheated samples showed a significant improvement over their reference counterparts. The optimized preheated sample showed an improved prior β-grain distribution with a circular morphology and thicker α laths within the even finer prior β-grain boundaries. Also, an overall increment of the c/a ratio of the HCP α has been observed, which yielded lattice strain relaxation in the localized grain structure. Furthermore, a less-profound surface roughness was observed in the preheated sample. The obtained microstructure with all these factors delivered a 10% improvement in its fatigue life with better mechanical strength overall.

## 1. Introduction

In recent years, additive manufacturing (AM) techniques have gained significant attention in a variety of industries ranging from aerospace to biomedical [1,2]. This is particularly due to their capability to fabricate complex geometries with controlled characteristics from CAD data without process planning [3,4,5]. Among the different AM techniques, laser powder bed fusion (LPBF) is the most adopted fabrication method, in which a high-power laser beam scans across a thin layer of deposited powder to melt and fuse it onto the previous layer [6]. A variety of materials can be processed using this technique, including but not limited to Ti-alloys, Al-alloys, Ni-alloys, Fe-alloys, Co-alloys, and Cu-alloys [6]. Ti-alloys are the most popular materials for lightweight engineering applications in corrosive environments in both aerospace and biomedical applications. Among the Ti-alloys, Ti-6Al-4V, which is also known as Ti grade 5, has the highest demand and it alone makes up almost half of the market share of titanium products fabricated in the world [7,8]. For example, when it comes to airframe parts such as the landing gear beam of a Boeing 747 [8] or the empennage of a Boeing 777 [9], about 80% of the volume of their titanium airframe parts are made up of Ti-6Al-4V [10]. Pertaining to jet engine parts such as the blisk (i.e., bladed disk) of the F-35 Lightning-II fighter or the fan disk/fan vane of a GE-90 aero-engine, more than 50% of the volume of their titanium parts are made up of Ti-6Al-4V [8].

Ti-6Al-4V is a great candidate for use in aerospace components that are exposed to cyclic loadings during their service, particularly because of its unique properties, e.g., its superior strength [11,12,13], exceptional corrosion resistance [14], its being lightweight and durable [15], having good fatigue performance [4,5,16,17], crack initiation resistance [18,19], and crack propagation resistance [20,21,22].

The combination of the fabrication capabilities of LPBF technologies with the superior material properties of Ti-6Al-4V promises intricate geometries for lightweight, robust engineering components. Even though the LPBF-fabricated Ti-6Al-4V offers an additional benefit of design freedom for complex geometries [23,24,25,26], it has been well documented in the literature that LPBF-fabricated parts demonstrate degraded fatigue properties when compared with those fabricated conventionally. The surface and the microstructure properties of the as-built condition of LPBF-fabricated Ti-6Al-4V promote an earlier crack nucleation compared to wrought Ti-6Al-4V [5] and result in a lower fatigue life [27]. This has been attributed to four major contributory factors: (i) the poor surface characteristics of LPBF-fabricated specimens [28], which are the result of the associated staircase effect [29,30,31], balling phenomena [32,33], and un-melted powder particles at the surface [34,35]; (ii) the metastable structure of LPBF-fabricated parts (i.e., the martensitic α′—phase [36], columnar prior-β grains [37], and anisotropic grain texture oriented along the build direction [38]), which is due to the involved directional solidification and cooling rates in the LPBF process; (iii) the inherent residual stress as a result of the rapid solidification and cooling rates of the process, the (iv) processing defects because of the interaction between the rapid laser scan and the loose powder bed, and the melt pool dynamics.

With the continuous advancement in the understanding of the LPBF-fabricated Ti-6Al-4V microstructure, some studies reported a fatigue life of laser-powder-bed-fusioned Ti-6Al-4V matching/converging with conventionally wrought Ti-6Al-4V [39] after the complementary processing of the microstructure. Post-processing options like a heat treatment (HT), hot isostatic pressing (HIP), and surface improvement are the most common applications. It is reported that hot isostatically pressed LPBF-fabricated Ti-6Al-4V can result in the same fatigue strength as the wrought counterpart [4,40]. The other common approach is the improvement of the surface finish. Chastand et al. [41] reported that additional surface polishing to hot isostatically pressed LPBF-fabricated Ti-6Al-4V improved the fatigue strength at 10^7^ cycles of the material, which is more than three times that of the stress-relieved (at 640 °C/4 h) as-built surface condition.

In addition to post-processing, there are process-induced microstructure improvement studies for enhancing the fatigue strength of LPBF-fabricated Ti-6Al-4V. These studies mostly evaluated the effect of the process parameters, building orientation, and the geometric factors during fabrication [3,42,43]. Hosseini et al. [44] reported the effect of various scanning strategies on the fatigue characteristics and the microstructure of LPBF-fabricated Ti-6Al-4V. It was concluded that the fatigue crack initiation did not happen at the defects which are located at the surface nor due to the high inherent residual stress. Xu et al. [45] studied the effect of the process variables and introduced a new approach: studying the focal offset distance (FOD) which maintains a close hatch spacing. Due to the limited adjustability of the FOD, this method was reported as being less practical without a complementary post-heat treatment [45]. Ali et al. [46] reported the effect of powder bed preheating during the fabrication of Ti-6Al-4V and it is evident that the preheating modified the martensitic decomposition and mechanical properties of the material. Owing to the requirement of an increased fracture toughness in those fields [47,48], a varied processing strategy has been studied for its effect on fatigue improvement and mechanical strength. 

In this study, an additional laser scan with different laser parameters was employed to preheat the powder particles along the surface immediately before the melting scan. Contrary to the electron-beam-melting (EBM) process which preheats the powder bed surface fully, an area-wise preheating laser scan is applied in the LPBF to the laser scan area at each layer here. This preheating laser scan was introduced to modify the microstructure of LPBF-fabricated Ti-6Al-4V. More innovatively, this strategy was only applied to the selected regions across the fatigue specimen. It is proven in the published studies that the surface characteristics and sub-surface microstructure define the fatigue behavior of LPBF-fabricated Ti-6Al-4V. Hence, a thickness of 0.5 mm along the specimen surface was arbitrarily selected for microstructure tailoring during the process. It was observed that the preheating laser scan tailored the microstructure of the sub-surface of the specimen by modifying the prior β grain morphology and the lath structure of the α-Ti. Further improvement in the surface roughness was noticed with the application of the layerwise bordered preheating laser scan. Results revealed that the proposed in situ local microstructure tailoring increases fatigue life up to 10% without any additional post-processing requirements with an improvement in the surface roughness.

## 2. Materials and Methods

Gas-atomized Ti-6Al-4V powder provided by EOS North America (Pflugerville, TX, USA) was used for fabrication. The powder particles exhibit a size range of 20–80 µm and consisted of 5.50–6.75% Al, 3.50–4.50% V, 0.20% O, 0.05% N, 0.08% C, 0.015% H, 0.30% Fe and balanced Tí (wt%) meeting the requirements of ISO 5832-3 [49], ASTM F1472 [50] and ASTM F2924 [51]. 

**Figure 1:**
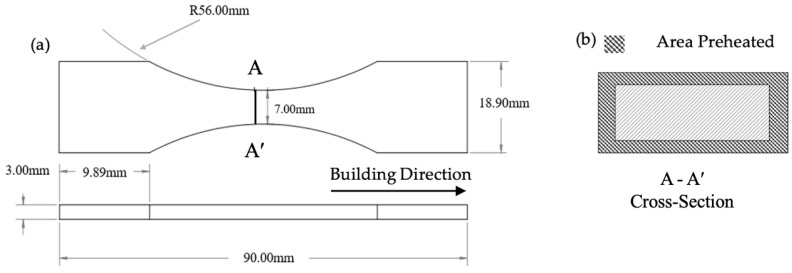
(**a**) Specimen size for both fatigue and tensile testing (ASTM E466-21 [52] specimens with tangentially blended: (i) The ratio of the specimen’s test section width to thickness should be between 2 and 6, (ii) the reduced area should preferably be between 19.40 mm^2^ and 645 mm^2^, and (iii) the radius of the blending fillets should be at least 8 times the specimen test section width). (**b**) Cross-section of the sample depicting the area that is preheated before the entire section is laser heated.

SolidWorks 21 software (Dassault Systems, Vélizy-Villacoublay, France) was utilized for both the specimen designs of the microstructure (20 mm × 6 mm × 6 mm) and the continuous radius (ASTM E466) [52] test specimens (Figure 1). Both specimens were fabricated longitudinally, aligning the longer side of the specimen to be perpendicular to the *z*-axis of the printer (building direction).

The specimens were fabricated in an EOS M290 3D printer (EOS GmbH, Electro Optical Systems, Krailling, Germany) equipped with a 1070 nm wavelength and 400 W Ytterbium fiber laser capability. The building plate was preheated to 80 °C and maintained the preheating temperature throughout the fabrication process to minimize the thermal gradients within the initial layers of the printing.

To quantize the laser scanning parameters’ effect, the energy density of the scanning strategies was calculated using Equation (1), where Ev represents the energy density (J/mm^3^), P is the laser power (W), v is the laser scanning speed (mm/s), t is the layer thickness (mm), and h is the hatch spacing (mm) [53].

(1) $$E_{v}=\frac{P}{v\cdot h\cdot t}$$

In this study, the novel approach of the multi-laser scan’s application was applied for preheating the powder particles only on the selected regions of the cross-section of the specimens with a lower laser scan power. As the samples were fabricated vertically along the tensile testing direction, the outer surface of the specimen was preheated using the respective energy densities before the melting scan was performed along the entire cross-section as shown in Figure 1b. Reference samples (non-preheated) were fabricated using only a melting laser scan with the default process parameters. The default laser parameters for melting were recommended by the manufacturer and defined as a laser power of 280 W, a scan speed of 1300 mm/s, a hatch spacing of 120 µm, and a layer thickness of 40 µm, resulting in an energy density of 44.87 J/mm^3^ [54]. The laser spot size was 100 µm and has a Gaussian-distributed energy. Other scanning parameters were defined as a stripe scanning strategy and a hatch angle of 47°. For the proposed layerwise local preheating application, two different laser energy density values were selected to evaluate the effect of the preheating laser scan on the microstructure of the LPBF-fabricated Ti-6Al-4V. First, a preheating energy density equivalent to 60% of the melting laser scan energy was selected to mimic the relationship between the melting temperature and the β-transus temperature of Ti-6Al-4V. (T_β_ = ~950–1050 °C and T_m_ = ~1600–1660 °C (T_β_ = ~0.6 T_m_)). Then, an energy density of 80%, higher than the preheating value of 60% but lower than that in the existing literature (100%), was chosen as the median. Amounts of 60% and 80% of the melting laser scan energy density were applied to study the effect of the preheating laser scan on the sub-surface microstructure. Except for the laser power, other process parameters were kept constant between the melting laser scan and the preheating laser scan. The preheating laser scan power was selected as 168 W and 224 W for the 60% and 80% energy densities of the melting laser scan, respectively. All specimens were carefully removed from the building plate using a wire electrical discharge machining (EDM) cutter (EDM Network, Inc., Sugar Grove, IL, USA).

Microstructure evaluations of porosity and grain morphology were examined using a scanning electron microscope (SEM). A Hitachi S-3000N variable pressure (Hitachi, Santa Clara, CA, USA) was utilized for microstructure imaging studies. SEM micrographs were processed using Image J 1.53 [55] software for a robust analysis (three different regions at magnification levels of 500× and 800×). The image processing includes the conversion of micrographs to RGB stack files for the threshold adjustment of the gray-scale images with an appropriate/adequate contrast to distinguish the microstructure features. The average data reported by Image J was used to quantize the porosity and the α/α’ lath thickness [56]. To prepare samples for SEM characterization, they were first cold-mounted into a mixture of epoxy resin and hardener followed by surface grinding and polishing using an E-prep 4™ polisher (Allied High-Tech Products Inc., Rancho Dominguez, CA, USA). The grinding process was conducted using silicon carbide (SiC) abrasive disks ranging from 360 to 1200 grit sizes. For the first step of the polishing, a DiaMat polishing cloth with a 1 µm diamond suspension was employed, then, for the final polishing step, a 0.04 µm colloidal silica on a Red Final C polishing cloth pad was applied to achieve scratch-free, mirror-like sample surfaces. Samples were rinsed with micro-organic soap and cleaned using isopropyl alcohol. Surface preparation was finalized with surface etching using Kroll’s reagent (1–3 mL of HF, 2–6 mL of HNO_3_, 100 mL of water) to delineate grain boundaries and phases.

A SEM micrograph was used to evaluate the fine microstructural features at a high magnification level. The resolution was enhanced using the ESRGAN [57] deep learning algorithm, followed by fine-tuning for contrast and brightness using the Python 3.10.0 OpenCV library [58].

A NORAN Systems 7 Thermo Fisher Scientific (Waltham, MA, USA) detector was used for electron diffraction spectroscopy (EDS) analysis to identify the chemical composition variance in the microstructure of the Ti-6Al-4V within the spectral resolution degradation of 3% up to 60% of the dead time. For EDS analysis, a point-and-shoot analysis was conducted for the preheated and core regions, respectively, at least three times to verify if there was any variability. One of them is added later in the manuscript.

A Bruker D8 Advance X-ray diffractometer (Bruker Corporation, Madison, WI, USA) was employed for the structural analysis of the fabricated material, utilizing a Cu K-alpha wavelength of 1.5406 Å, a current of 40 mA, and a voltage of 40 kV at room temperature with 0.05° step intervals. Measurements were conducted at a speed of 1/step, with 2θ ranging from 20° to 80°. X-ray diffraction (XRD) analyses were studied to identify the phases and grain texture of each sample’s microstructure. Using Bragg’s law [59], the lattice parameters of a and c were calculated. As shown in Equations (2) and (3), inter-planar spacing d and lattice parameters were obtained in which h, k, and l indicate Miller indices:

(2) λ=2dsin(θ)

(3) $$\frac{1}{d^{2}}=\frac{4}{3}\left(\frac{h^{2}+hk+k^{2}}{a^{2}}\right)+\frac{l^{2}}{c^{2}}$$

In order to investigate the microstrain condition of the microstructure within the reference and preheating laser scan application, the Williamson-Hall (W-H) analysis was utilized [60], which is illustrated in Equation (4). In this equation, β, D, and ε are the peak broadening, crystallite size, and microstrain, respectively. To calculate the microstrain, βcosθ was plotted versus 4sinθ in which the slope gives the amount of microstrain within the microstructure. The peak broadening of related XRD peaks is considered to be the full-width-at-half-maximum (FWHM) obtained by fitting a calculated profile on the real XRD data using OriginPro 8.5 software. Si standard reflection (0.0013 rad) [61] was subtracted before strain analysis using the W-H method. Furthermore, it needs to be noted that θ can be obtained from the peak positions achieved by the multi-peak fitting analysis in which the wavelength (λ) and k are 1.5406 Å and 0.94, respectively.

(4) $$\beta cos\theta =\frac{\lambda k}{D}+4\varepsilon sin\theta$$

Mechanical testing for both tensile and fatigue tests were conducted using a Shimadzu EHF E-Series (100 kN) with a 4830-servo controller (Shimadzu Scientific Instruments, Inc., Missouri City, TX, USA). Test equipment are coupled with a digital image correlation (DIC) system (Correlated Solutions, Inc., Irmo, SC, USA) for precise strain measurement. The displacement and strain at the surface were monitored by tracking the light intensity patterns of high-contrast speckles on the specimen surface. Two Grasshopper GS3-U3-23S6M (FLIR Systems, Inc., Santa Barbra, CA, USA) CCD cameras with 2.3 MP each, and a pixel array of 1920 × 1200, were utilized for image capturing during testing. The correlation of the test equipment data and the image processing were performed using VIC-3D-9^®^ software (Correlated Solutions, Inc., Irmo, SC, USA). The loading rate for tensile testing was determined to be 1.2 mm/min according to the recommended testing standard for LPBF-fabricated Ti-6Al-4V [62]. The stress-controlled fatigue test parameters were a 5.232 kN applied load under a 50 Hz frequency and an R = 0.1. In order to verify the variability in the tensile test and fatigue results, three specimens were tested in each of the preheating cases as well as reference specimens for the tensile test and fatigue results. So, a total of 18 Specimens were fabricated.

The surface roughness of the fatigue specimens was measured by a Mitutoyo Surfest SJ-210 profilometer (Mitutoyo America Co., Aurora, IL, USA). Roughness measurements were conducted following ISO 21920-2 [63] at least three times and the average value was used for the comparison and the evaluation of the applied layerwise locally preheating laser scan of LPBF-fabricated Ti-6Al-4V. 

## 3. Results

### 3.1. Microstructure Modification

**Figure 2:**
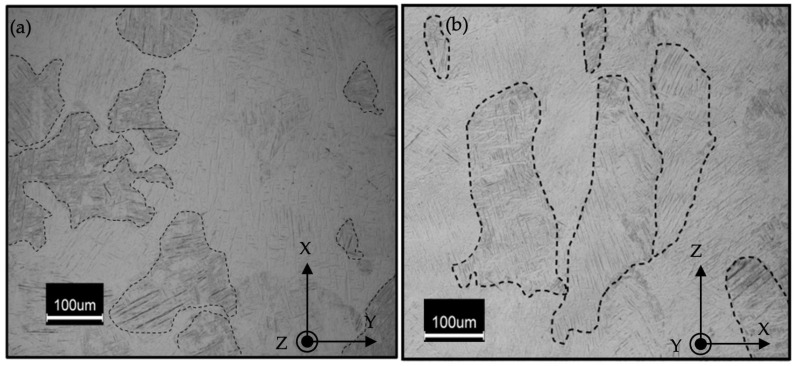
Prior β-grain boundaries have been affected by the directional cooling; (**a**) a microstructure image of the building (XY) plane shows the randomly distributed polygonal shape of the prior β-grain boundaries, and (**b**) elongated prior β-grains through the building direction (ZX Plane).

It was observed that the reference sample without any preheating laser scan has randomly distributed polygonal shaped prior β grains along the XY plane (perpendicular to the building direction); however, prior β grains were elongated and oriented vertically along the building direction, which is consistent with previous studies [7]. Darker regions in Figure 2 depict the prior β grains. 

**Figure 3:**
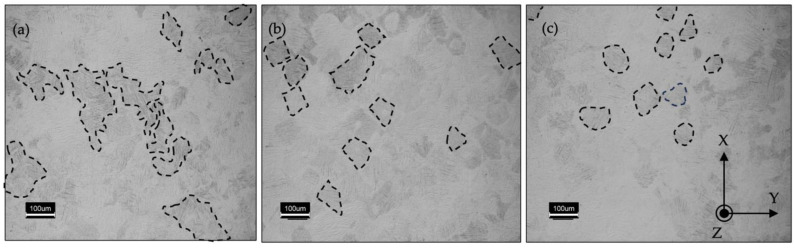
Prior β-grain boundary transformation with applied preheating energy densities; (**a**) Reference, (**b**) E_v_ = 26.92 J/mm^3^, and (**c**) E_v_ = 35.90 J/mm^3^.

Further investigation into prior β-grains revealed that the energy level introduced to the microstructure during the preheating laser scan has an effect on the grain morphology. Results unveiled that prior β-grain boundaries changed from being random and polygonal to being quadratic and finally to a circular shape with the increase of the energy density. Figure 3 depicts the prior β-grain boundaries of the reference, and those following preheating with 26.92 J/mm^3^ and 35.90 J/mm^3^.

**Figure 4:**
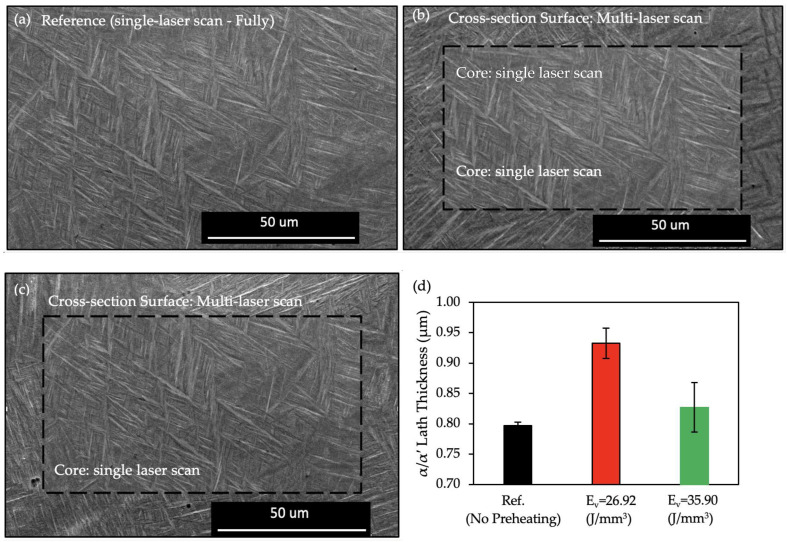
α/α′ Lath structure of the reference sample (no preheating) (**a**), the surface microstructure after preheating with an E_v_ = 26.92 J/mm^3^ and the core microstructure of the specimen (**b**), the surface microstructure after preheating with an E_v_ = 35.90 J/mm^3^ and the core microstructure of the specimen (**c**), and the thickness of the lath structure of the specimens at the applied preheating regions (**d**).

Additionally, the modification of the α-phase was evaluated and it was noticed that the preheating laser scan adjusts the lath size of the LPBF-fabricated Ti-6Al-4V. Figure 4 depicts the micrographs of the reference and of the preheated regions of the specimens. Lath thickness was also quantized by image processing to gain a better understanding of the effect of the preheating energy density on the microstructure. 

Figure 4 plots the average lath thickness of the reference sample as 0.797 ± 0.006 µm. Both preheating energy densities increased the lath thickness of the α/α′ phase and it was measured to be 0.933 ± 0.025 µm and 0.827 ± 0.041 µm for E_v_ = 26.92 J/mm^3^ and E_v_ = 35.90 J/mm^3^, respectively. The thickness variation of the α/α′ phase between the reference (no preheating) specimen and the core regions of the preheated sample, where there was no preheating laser scan, was insignificant. 

**Figure 5:**
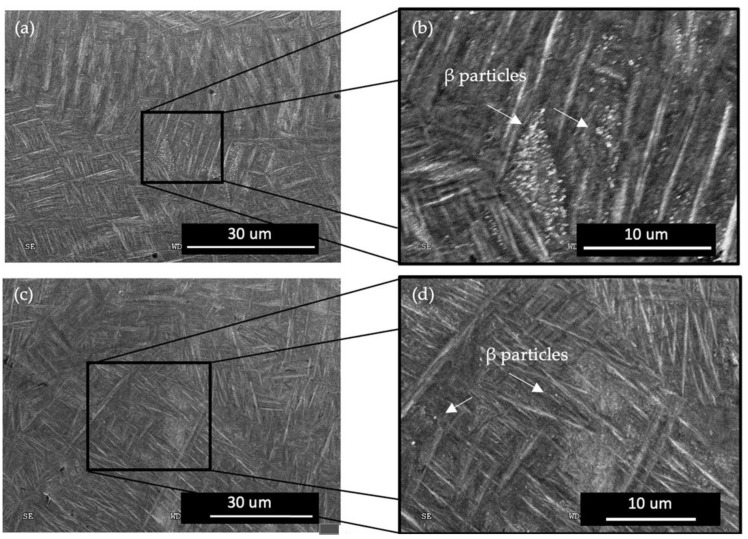
β phase particles in the microstructure of the preheated regions with an E_v_ = 35.90 J/mm^3^ preheating energy at low magnification level (**a**) and high magnification level (**b**), β phase particles in the microstructure of the reference (no preheating) specimen at low magnification level (**c**) and high magnification level (**d**).

Micrographs at higher magnification levels (≥5000×) exposed β grains at room temperature (RT) in the microstructure of the preheated region with an E_v_ = 35.90 J/mm^3^. It was observed that the β-grain regions at RT were not significant in the other specimens (Figure 5).

**Figure 6:**
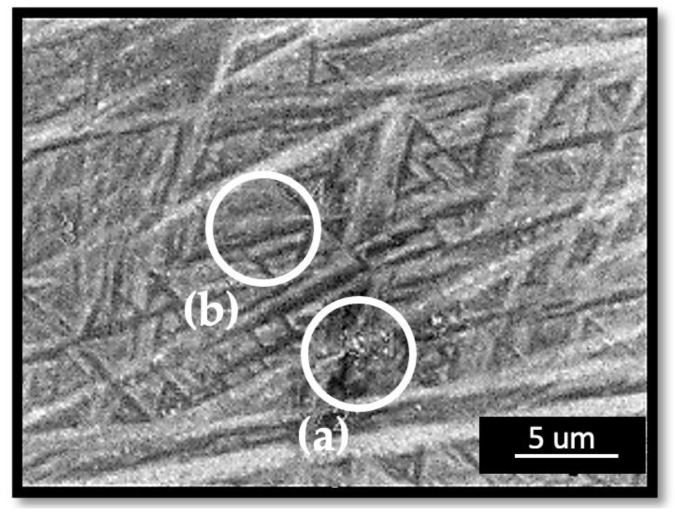
EDS analysis at a ×5000 magnification level of the LPBF-fabricated Ti-6Al-4V’s (**a**) vanadium-rich region (bright dots) and (**b**) the Ti-6Al-4V material’s α + β-phase region.

EDS analysis was conducted to examine the chemical composition of the bright regions. It was observed that the bright regions had higher vanadium contents, vanadium being the β-phase stabilizer element of the Ti-6Al-4V alloy. The chemical composition in Figure 6a was measured to be 5.30% ± 0.08 Al (wt.%), 3.13% ± 0.01 V (wt.%), and 91.57% ± 0.07 Ti (wt.%), and in Figure 6b, it was measured to be 5.32% ± 0.11 Al (wt.%), 3.09% ± 0.01 V (wt.%), and 91.57% ± 0.01 Ti (wt.%).

The internal defects of the microstructure were quantized during microstructure characterization by image processing. The initial microstructure of the LPBF-fabricated Ti-6Al-4V was measured to be 0.47% ± 0.015. The porosity level of the microstructure was measured to be 0.041% ± 0.011 and 0.030% ± 0.03 for the preheating laser scans of E_v_ = 26.92 J/mm^3^ and E_v_ = 35.90 J/mm^3^, respectively.

### 3.2. Crystallography

**Figure 7:**
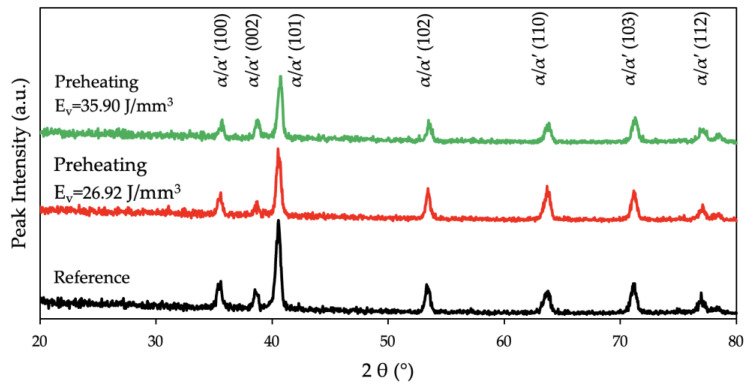
XRD patterns of the reference (no preheating) sample (black) and preheated samples with an E_v_ = 26.92 J/mm^3^ and an E_v_ = 35.90 J/mm^3^ in red and green, respectively.

Regarding the crystallographic characterization of the material conducted through XRD analysis, Figure 7 depicts the diffraction patterns of the reference and of the preheated samples. The highest peak intensity was observed at (101) for the reference and the preheated samples. The reference sample had the highest intensity and the preheated sample with an E_v_ = 35.90 J/mm^3^ had the lowest intensity at (101). Additionally, a reduction at the full width half maxima (FWHM) with the application of the preheating laser was observed.

**Figure 8:**
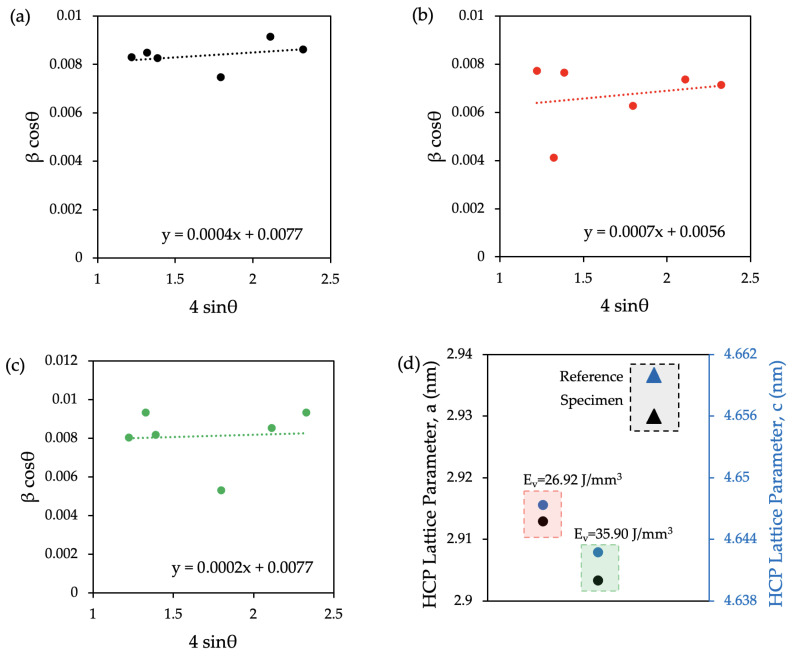
W-H analysis plot: the microstain values derived from the slope of the fitted linear trendline of the (**a**) reference sample, (**b**) preheated specimen at E_v_ = 26.92 J/mm^3^, (**c**) preheated specimen at E_v_ = 35.90 J/mm^3^, and the (**d**) lattice parameters of the reference and the preheated samples.

The XRD data quantized through the W-H model and the microstrain value are plotted in Figure 8. Results did not show a distinct variation between the reference sample with the initial condition and the surface-preheated regions of the specimens. It was observed that plotted graphs exhibit a positive slope which is the result of the tensile strain.

In addition to the W-H analysis, the lattice parameters were calculated according to Bragg’s law. The c/a ratios of the reference sample, the preheated sample with an E_v_ = 26.92 J/mm^3^, and with an E_v_ = 35.90 J/mm^3^ were calculated as 1.5904, 1.5954, and 1.5990, respectively. A lattice strain at the layerwise surface-preheated regions was observed in the microstructure. The lattice parameters of the α-Ti (Hexagonal Closed Pack—HCP) are plotted in Figure 8d. The results show that the preheating laser scan decreased the lattice parameters of the α-Ti.

### 3.3. Surface Properties

**Figure 9:**
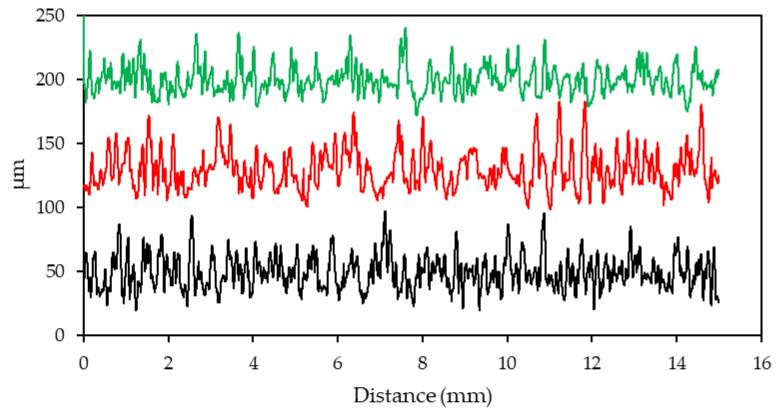
The effect of the layerwise surface-preheating laser scan on the surface roughness of the specimen. (reference—no preheating laser scan: black, preheating laser scan at E_v_ = 26.92 J/mm^3^: red, and preheating laser scan at E_v_ = 35.90 J/mm^3^: green).

The surface roughness profile of the specimens was plotted in Figure 9. The preheated specimen with an E_v_ = 26.92 J/mm^3^ demonstrated the roughest surface among the other specimens. The finest surface finish was observed on the preheating specimen with the laser energy density of E_v_ = 35.90 J/mm^3^. The average roughness of the reference sample without the preheating laser scan was measured as Ra = 10.4 ± 0.6 µm. The finer surface that was observed in the preheated sample (E_v_ = 35.90 J/mm^3^) was Ra = 8.7 ± 0.9 µm. The other preheated specimen (E_v_ = 26.92 J/mm^3^) exhibited a roughness value of Ra = 11.6 ± 0.8 µm.

### 3.4. Mechanical Properties

**Figure 10:**
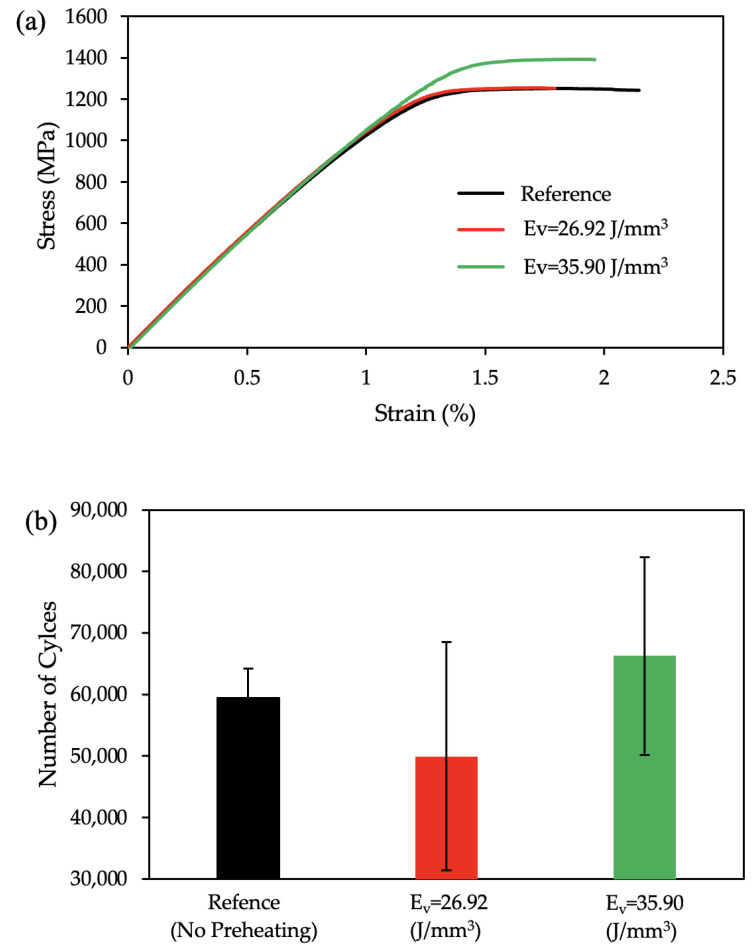
The mechanical response of the microstructure scanned with a surface-preheating laser. (**a**) Tensile test plot, (**b**) Fatigue test results (reference—no preheating laser scan: black, preheated with E_v_ = 26.92 J/mm^3^: red, preheated with E_v_ = 35.90 J/mm^3^: green).

The tensile test results are plotted in Figure 10a, where a black line represents the reference sample without any preheating laser scan, and the red and green lines represent the preheated samples with an E_v_ = 26.92 J/mm^3^ and an E_v_ = 35.90 J/mm^3^, respectively. The measured Ultimate Tensile Strength (UTS) values were 1242 Mpa, 1253 Mpa, and 1390 Mpa for the reference, the specimen preheated with an Ev = 26.92 J/mm^3^, and the specimen preheated with an E_v_ = 35.90 J/mm^3^, respectively. The preheated specimen with an E_v_ = 35.90 J/mm^3^ showed a significant enhancement in mechanical strength (UTS) of about 12% above the reference, whereas the specimen with an E_v_ = 26.92 J/mm^3^ had a similar strength with a lower elongation. Compared to the reference sample, both the preheated samples exhibit a lower elongation. The observed maximum strain during the tensile test for the specimens, the reference, preheated with an E_v_ = 26.92 J/mm^3^, and preheated with an E_v_ = 35.90 J/mm^3^, were 2.14%, 1.79%, and 1.96%, respectively.

Fatigue test results were also plotted in Figure 10b. It was observed that the applied layerwise surface preheating improved the total number of cycles during the fatigue test for the specimen with a preheating of an E_v_ = 35.90 J/mm^3^. However, the average fatigue life of the preheating laser scan application at an E_v_ = 26.92 J/mm^3^ exhibits a lower lifetime compared to the average of the reference specimens without any preheating application.

## 4. Discussion

### 4.1. Preheating Laser Scan Energy Management to Control Defect Formation

**Figure 11:**
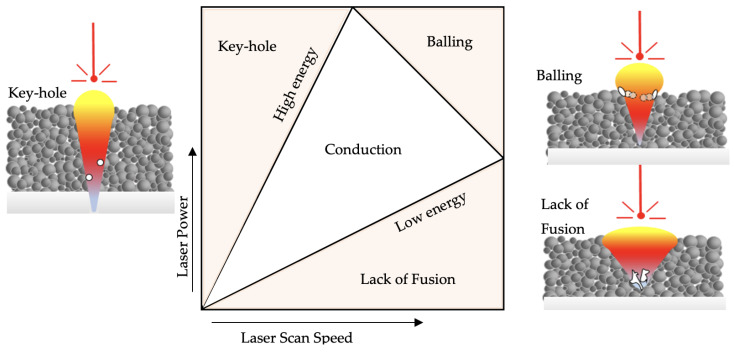
Melting mode according to the applied laser scan energy and the schematic view of the defects’ (white regions) formation for the melting modes.

One of the objectives of this study is to regulate the cooling and solidification rates to modify the LPBF-fabricated Ti-6Al4V material’s microstructure. A slower cooling can be achieved by using a higher energy input; however, if the high energy is delivered to the powder bed at once during the melting laser scan, the melt pool transforms from conduction to key-hole mode [64]. In key-hole mode, the excessive energy at the laser source’s center exceeds the evaporation temperature of the Ti-6Al-4V. Consequently, this excessive energy at the center of the Gaussian laser beam reshapes the melt pool hydrodynamics with a deeper melt pool and causes gas porosities [65]. Thus, the authors consider it to be a boundary condition during the process design and introduce the total energy to the powder bed in a sequence to avoid the defect formation due to key-hole mode (Figure 11).

A preheating laser scan was introduced to the powder bed selectively to increase the total energy input, decreasing the cooling and solidification rates compared to those of the single melting laser scan (the default application of the reference sample). The multi-laser scan approach has been studied previously [66,67]. However, their primary focus was developing a better understanding of the re-melting effect on the material’s microstructure and mechanical response by using equivalent laser parameters. The effect of the preheating laser scan on the full cross-section was presented and revealed that the preheating laser scan improves the mechanical strength by reducing the elongation [56].

In the present study, the authors have taken the previous research a step further by selectively applying the preheating laser scan to enhance the surface strength while minimizing the reduction in elongation which is discussed in the following sections. The relation between the β-phase transformation temperature and the melting point of the Ti-6Al-4V (Tβ ≈ 0.6 Tm) has been replicated in the selection of energy for the preheating laser scan which promotes the grain structure recrystallization and relieves the internal residual stress. For the second preheating energy level, the effect of the 100% laser scan energy for a multi-scan approach is reported. So, the authors have selected the median between 60% and 100% as the second preheating energy value for investigation in the layerwise selectively applied preheating laser scan where the literature lacks sufficient information. During the application of both preheating energy values, however, there was a partial melting between contact points of some powder grains after the fully melting preheating did not occur consistently with the laser sintering process, especially for the energy level of Ev = 35.90 J/mm^3^ [68].

### 4.2. Microstructure Refinement in Ti-6Al-4V Using LPBF

It is known that the overall morphology of the prior β-grain boundaries varies due to the directional cooling and solidification in the LPBF process [69]. The microstructure of the specimens demonstrates consistent phenomena in both the reference and the preheated regions based on the electron micrographs. The additional energy introduced to the microstructure during the preheating laser scan did not affect this aspect; however, it was observed that the preheating laser scan modifies the prior β-grain structure along the XY plane. Figure 3 reveals the response of the grains with respect to the applied preheating laser scan energy density. It was discovered that a low-energy preheating laser scan led to a quadratic grain structure where a higher preheating laser scan energy ended in a circular prior β-grain structure. This response of the prior β grain is in parallel with that of a previous study [70]. Comparing the geometry of the prior β grains, surface area degradation was observed with the introduction of the preheating laser scan. Similar grain refinement of the prior β-grain structure was studied by Zou et al. [70] where the effect of the prior β-grain structure on the tensile properties of Ti-6Al-4V was reported. Authors modified the prior β-grain structure using a post-HT and the results revealed that a finer prior β-grain structure significantly improved (≥3 times) the elongation characteristic of the LPBF-fabricated Ti-6Al-4V [70].

It is well understood that the α/αˈlaths grow along ∼45° of the prior β-grain boundaries, which is a result of the favorable Burgers orientation relationships [71,72,73]. Figure 4 depicts the α/αˈlaths mostly oriented towards ∼45° with respect to the specimens’ edges along the XY plane. It is demonstrated that the layerwise surface-preheating laser scan increased the average lath thickness significantly with the application of an E_v_ = 26.92 J/mm^3^. However, the impact of the surface preheating on the lath thickness upon the application of an E_v_ = 35.90 J/mm^3^ was not as noticeable as that of the application of an E_v_ = 26.92 J/mm^3^. The lath thickness increase with the introduction of the preheating scan can be rationalized with the reduction of the cooling rate with the higher energy input compared to the reference sample [56]. When it comes to the comparison of the α/αˈlaths between the two preheating applications, the cooling rate itself was not enough to understand the reaction of the microstructure. A more sophisticated approach was required where the β → α decomposition was deeply investigated to understand the divergence between the applied preheating energy densities. Etesami et al. [74] reported that at very high cooling rates, β transforms to α’. At relatively lower cooling rates, it decomposes to secondary α with a rod-shaped β between the α boundaries. The authors concluded that a further decrease in the cooling rate resulted in the β’s decomposition to thick secondary α lath at the boundaries. This information sheds light on the thicker lath structure at the lower energy input (E_v_ = 26.92 J/mm^3^) during the preheating laser scan. It was assumed that the thicker lath structure at the E_v_ = 26.92 J/mm^3^ preheating microstructure consists of rod-shaped β grains and that they could have been counted towards α/αˈlaths during image processing. Further evaluation of the microstructure features will be conducted in the upcoming studies.

Micrographs at higher magnifications verified the β → α decomposition disparity between the applied energy densities during the preheating laser scan of the building process in the present study. Figure 5b demonstrates the bright particles in the microstructure at RT over higher magnifications. The reference sample had very few white particles compared to the preheated sample at an E_v_ = 35.90 J/mm^3^. Some studies reported them to be a β phase at RT [56,74], whereas Liu et al. [75] concluded similar such bright particles to be Ti_3_Al precipitates. The oversaturated vanadium atoms were expelled from the α phase during the long-term aging process and Ti_3_Al started to precipitate when the content of the aluminum surpassed the critical solid solubility of the Ti_3_Al. Additionally, Pantawane et al. [76] reported these bright regions to be the Al-rich regions and claimed that etching using Kroll’s etchant corroded the β phase faster where the darker regions were vanadium-rich. However, their methodology could not disclose a chemical composition variation due to the limited sensitivity of their instruments [76]. Liu et al. [75] confirmed the Al precipitates via XRD patterns. With respect to such varying instances, in the present study, XRD analysis was conducted (Figure 7) and Ti_3_Al (α_2_) was not detected in the diffraction patterns, which concludes the discussions of the bright particle region being the β particles [46,77,78,79]. The results of the EDS analysis supported this assertion, as the region with the bright particles had a higher amount of vanadium which stabilizes the β-phase. In Figure 6a, the vanadium content was higher at 3.13% ± 0.01 (wt.%) compared to the region without bright particles in Figure 6b, which had a vanadium content of 3.09% ± 0.01 (wt.%).

It is evident that a higher energy input during the layerwise surface-preheating laser scan transformed the amount of β-particle precipitation and its distribution in the LPBF-fabricated Ti-6Al-4V microstructure (Figure 5). In parallel to this finding, Etesami et al. [74] reported that increasing the preheating temperature of the building plate to 200 °C during the LPBF of Ti-6Al-4V affected the β particles in the microstructure. It was reported that increasing the build plate’s temperature promotes the formation of β, compared to the relatively low preheating temperature and the as-built condition [74].

The quantized data from the XRD patterns are plotted in Figure 8. A previous study reported the effect of thermal processing on the HCP lattice structure of the LPBF-fabricated Ti-6Al-4V [56]. Due to the limited application area of the preheating laser scan in the specimen cross-section, the crystallographic modification of the material was not observed at the same level as the fully preheated microstructure [56,80]. Additionally, no trend was disclosed in the microstrain value between the different preheating energy densities. All the specimens exhibit a positive slope which indicates the presence of the tensile strain [81]. However, the lattice parameters, both a and c, were decreased in both preheating laser scan applications. It is evident that the c/a ratio increased with the applied preheating laser scan. To the authors’ knowledge, this increment is related to the lattice strain’s relaxation [82]. Previous studies investigated the lattice’s deformation and reported the mechanical response of the material [83,84]. The effects on the microstructure can be correlated to the mechanical properties as follows.

### 4.3. Functionally Graded Microstructure for the Enhanced Fatigue Behavior of LPBF-Fabricated Ti-6Al-4V

**Figure 12:**
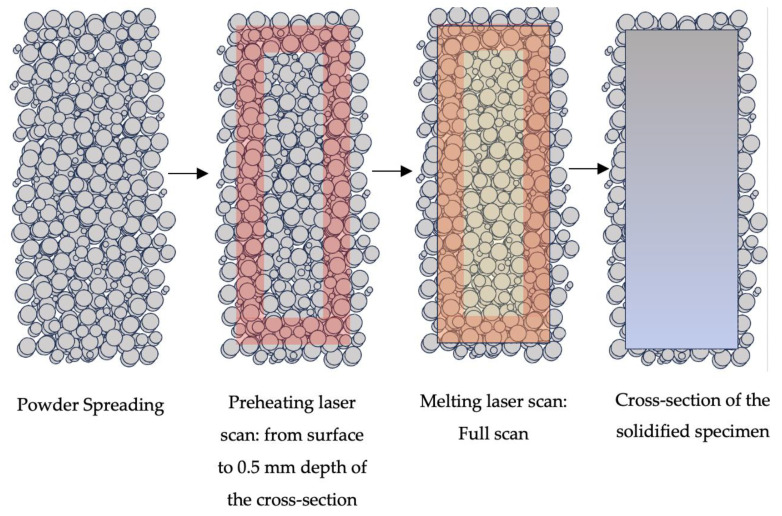
Schematic view of the in situ thermal processing steps to generate a functionalized microstructure and enhanced surface characteristics for a better fatigue behavior of LPBF-fabricated Ti-6Al-4V.

This study explores a novel approach to enhancing the fatigue strength of LPBF-fabricated Ti-6Al-4V by functionalizing the microstructure through controlled cooling rates at selected regions, a method not previously investigated. Published studies aiming to create a functionalized microstructure for specific mechanical properties modified the microstructure of the Ti-6Al-4V using additional elements or compounds [85,86,87]. Due to the discrepancy between the melting characteristics of the main material (Ti-6Al-4V) and those of the additive elements and compounds, these applications are challenging in terms of their process-induced defects. The energy input during the fabrication requires precise control and adjustment. Moreover, the homogenous particle distribution of the additional elements and compounds during powder spreading is difficult to standardize and an inhomogeneous element distribution was reported [86]. The proposed methodology (Figure 12) modified the microstructure and surface characteristics due to the applied region of the specimens while keeping the chemical homogeneity. Furthermore, the process defects reported in the previous studies that require some effort to overcome were not a concern in the presented study [87].

The enhancement in the mechanical strength of the E_v_ = 35.90 J/mm^3^ specimen when compared with that of the E_v_ = 26.92 J/mm^3^ specimen could be attributed to both the prior β-grain refinement and the improved surface finish values. The effect of the finer prior β grain is discussed in detail in the previous section. The rougher the specimen, the lower its strength. Greitemeier et al. [88] observed that the EBM specimen gave rise to Ti-6Al-4V samples with rougher surfaces in comparison with the laser-powder-bed-fusioned Ti-6Al-4V samples, which correlated negatively with its tensile strength. An investigation made by Liu et al. [89] over the α lath thickness vs. deformation ability proved that a higher lath thickness induces easier deformation due to an increased density of dislocations. From Figure 4d it can be proven that the lath thickness increase in the E_v_ = 26.92 J/mm^3^ sample had a lower strength compared to that of the E_v_ = 35.90 J/mm^3^ sample.

Figure 9 shows that the preheating laser scan with the energy level of E_v_ = 35.90 J/mm^3^ improved the surface finish of the LPBF-fabricated Ti-6Al-4V significantly. The average roughness of the reference sample was improved by 16% with the application of layerwise surface preheating (E_v_ = 35.90 J/mm^3^). This finer surface is a result of the partial melting during the preheating laser scan. Applied energy caused partial melting and agglomerated some particles which impeded the scattering of the powder particles during the melting laser scan. Khorasani et al. [90] reported that a higher total energy input caused a wider melt pool during the melting scan which overflowed to the outer powder particles and led to a reduction in the surface quality. However, the total energy was higher in the preheating laser scan (E_v_ = 35.90 J/mm^3^) since the energy was not applied at once, and the results proved the surface quality was improved. On the contrary, the preheating laser with the energy density of E_v_ = 26.92 J/mm^3^ weakens the surface finish, which can be justified by the effect of the preheating energy level on the melt pool during the melting scan, and a future study will address this and seek to find the optimum surface preheating laser scan strategy.

The primary aim of this study is to present the proof of the concept of modifying the microstructure of the selected regions in Ti-6Al-4V materials during LPBF fabrication without using alloying elements. Future studies could benefit from additional characterization techniques such as transmission electron microscopy (TEM) and additional mechanical testing. In the present study, it is rational to rely on the EDS analysis for the β phase (bright particles) at RT according to the vanadium content of the region because the variant is higher than the tolerance of the EDS technique (±0.01%). Also, additional information could be generated on the β → α + β decomposition by using TEM on the application of a preheating laser scan in LPFB-fabricated Ti-6Al-4V. While the results of the mechanical testing clearly indicate the effect of the applied thermal process during fabrication, future studies such as micro-hardness and four-point bending test evaluations would add more strength to this. Additionally, the authors assumed there to be a partial melting during the applied preheating, especially at the energy level of E_v_ = 35.90 J/m^3^ (80% of the melting laser scan power), in the context of the existing literature. There might be a benefit to the literature if this amount is quantized with an in situ thermal process monitoring system.

## 5. Conclusions

- Microstructural analysis demonstrates that the prior β-grain boundaries differ during the LPBF process. By performing preheating, the prior β-grain structure is modified along the XY plane. Low-energy preheating results in a quadratic grain structure and high-energy preheating, on the other hand, produces a circular prior β-grain structure.
- The $\alpha /\alpha^{\prime }$ lath thickness is increased by performing preheating during LPBF. This increment is related to the reduction in the cooling rate resulting from the higher energy input in the preheated samples compared to the reference ones.
- By performing preheating, the β→α decomposition disparity is promoted, and some brighter regions are formed within the microstructure. Furthermore, the number of these brighter regions is enhanced by increasing the energy density in the preheated specimens. Further studies regarding these particles showed that they represent the β phase in the microstructure at RT.
- Results of the W-H analysis assert that the microstrain does not follow any trend between the different preheating regimes; however, its value reveals the presence of the tensile strain within the microstructure. Also, it is worthwhile to note that the lattice parameters of a and c are reduced by performing preheating. Quite contrary to this, the ratio of c/a is enhanced as expected due to the lattice strain relaxation.

## Data Availability

Data are contained within the article.
